# INTEREST GROUP SESSION—BEHAVIORAL INTERVENTIONS FOR OLDER ADULTS: ADDRESSING CLINICAL SYMPTOMS OF DEMENTIA: PRELIMINARY RESULTS FROM DEMENTIA BEHAVIOR TRIAL

**DOI:** 10.1093/geroni/igz038.648

**Published:** 2019-11-08

**Authors:** Katherine A Marx, Laura N Gitlin, Joseph E Gaugler

**Affiliations:** 1 Johns Hopkins University, Baltimore, Maryland, United States; 2 Drexel University, Philadephia, Pennsylvania, United States; 3 University of Minnesota - School of Public Health, Division of Health Policy and Management, Minneapolis, Minnesota, United States

## Abstract

Currently, just under six million people living in America are diagnosed with Alzheimer’s disease or related dementia. Most people with dementia live in a community setting and are cared for by a family member. Persons living with dementia almost universally experience behavioral and psychological symptoms (BPSD), such as agitation, aggression, and rejection of care as well as functional dependence. These symptoms are related to negative outcomes for both the person living with dementia and the family caregiver. Prior research shows that nonpharmacologic interventions such as meaningful activities, education, and multicomponent interventions have promise in managing behaviors. This symposium focuses on preliminary outcomes from the Dementia Behavior Study (DBS), a Randomized Control study that examined the effect of the Tailored Activity Program (TAP) in a community setting on BPSD and functional dependence in persons living with dementia, and caregiver wellbeing (e.g. depression, burden, perceived change). Gitlin et al will present outcomes of the primary aim (BPSD) and secondary aims (functional dependence and caregiver wellbeing) of the DBS. Pizzi et al explore the cost analysis of the TAP intervention versus the active control group. Scerpella et al describe the alerts and adverse events that were associated with the DBS. Marx et al present the relationship between race and caregiver readiness to participate in TAP. Regier et al explore the BPSD Rejection of Care and the association to caregiver burden. Tailoring interventions, such as activities may improve quality of life for both the person with dementia and the family caregiver.

